# Supplementary figures and images for: Hygienic behaviour selection via freeze-killed honey bee brood not associated with chalkbrood resistance in eastern Australia
Source: PLoS One. 2018 Nov 14;13(11):e0203969. doi: 10.1371/journal.pone.0203969 (PMC6235251; doi:10.1371/journal.pone.0203969)

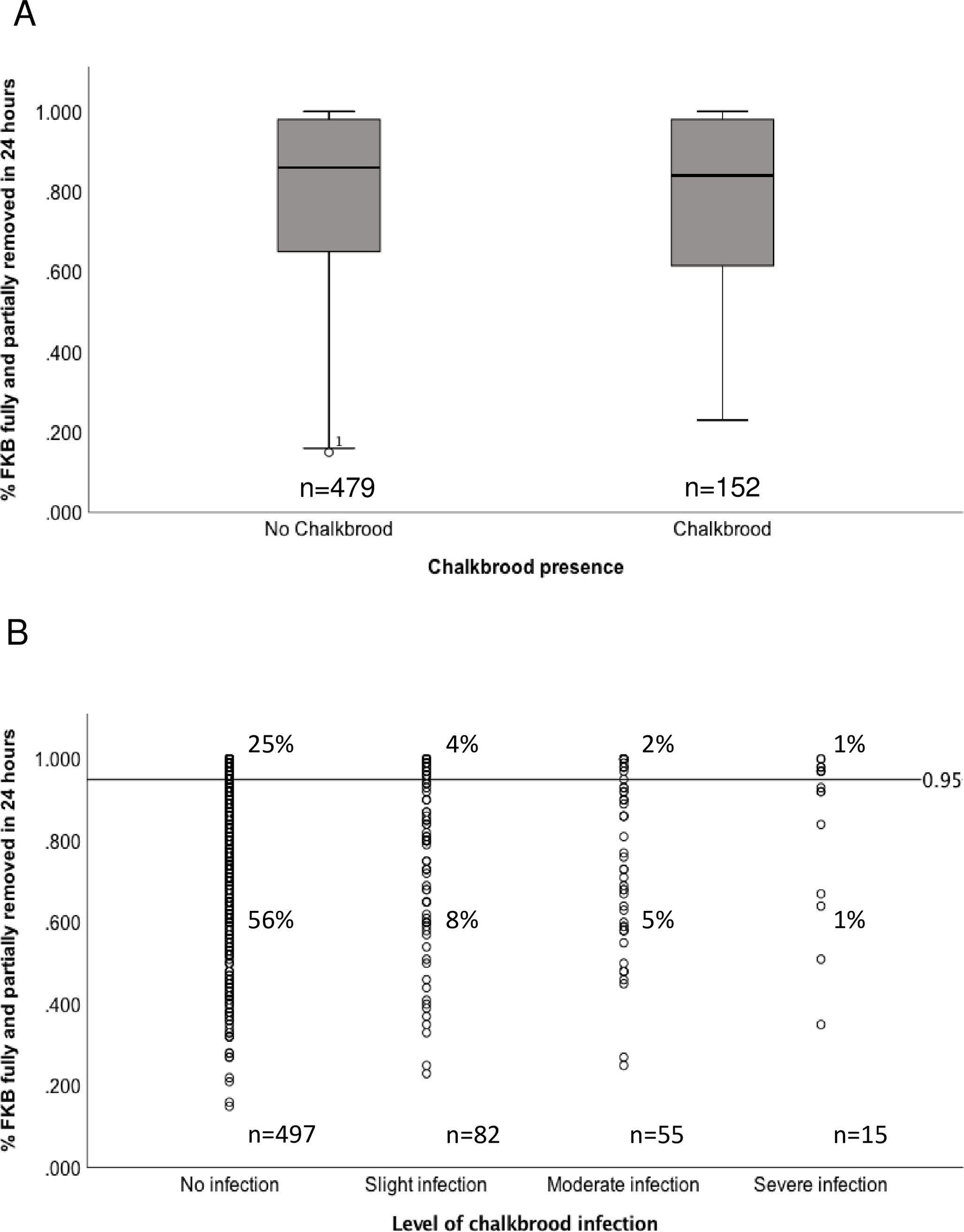

Supplement: S1 Fig — Hygienic behaviour as determined by the liberal FKB test not a predictor of chalkbrood infection (A) hygienic behaviour and chalkbrood infection (B) Severity of chalkbrood infection and liberal hygienic behaviour. Percentages are out of 649 colonies. (TIF) [file pone.0203969.s001.tif]

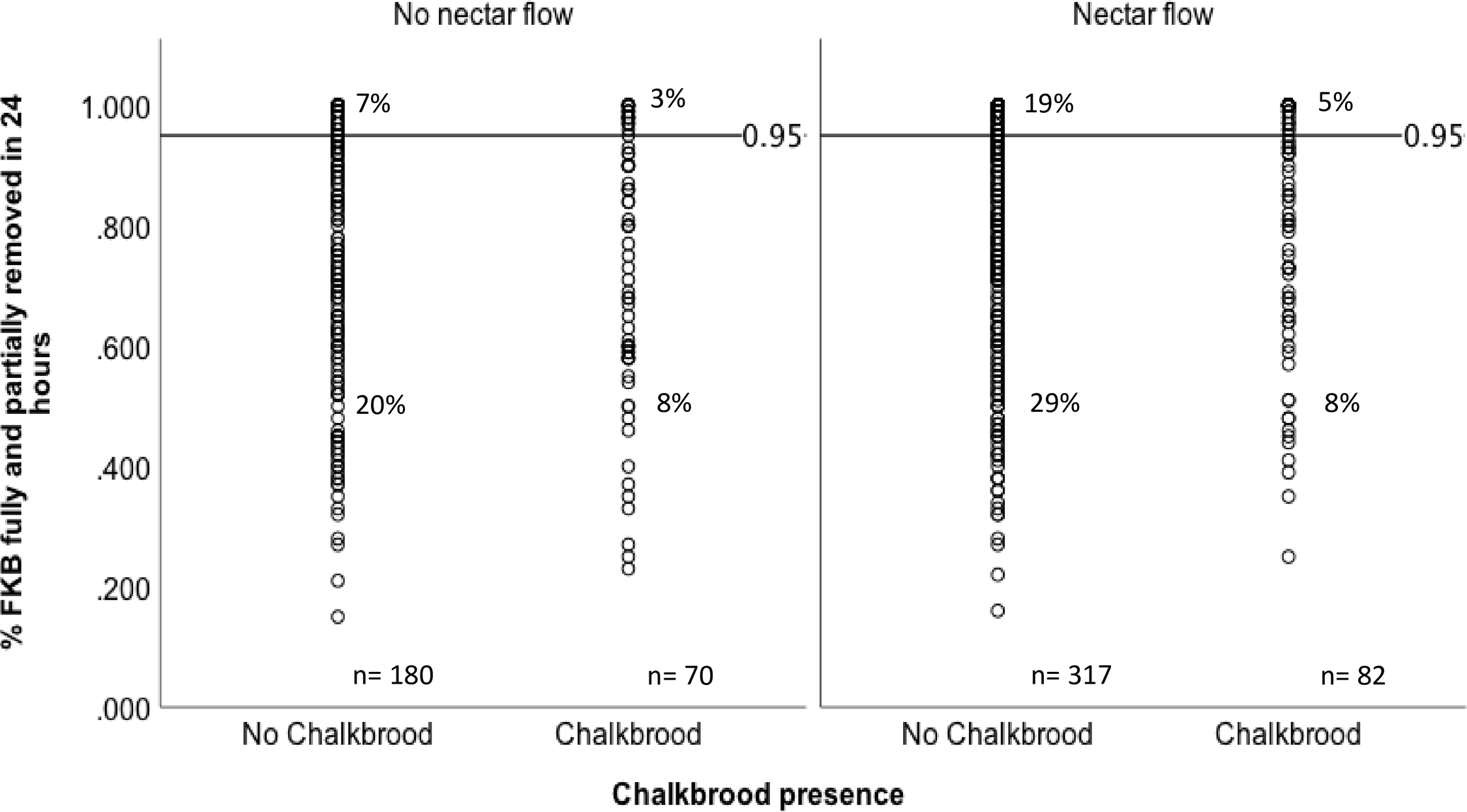

Supplement: S2 Fig — (TIF) [file pone.0203969.s002.tif]
