# Supplementary material for: Hygienic behaviour selection via freeze-killed honey bee brood not associated with chalkbrood resistance in eastern Australia
Source: PLoS One. 2018 Nov 14;13(11):e0203969. doi: 10.1371/journal.pone.0203969 (PMC6235251; doi:10.1371/journal.pone.0203969)
Supplement: S1 Table — (DOCX) [file pone.0203969.s004.docx]

| **S1 Table. Summary of logistic regression analysis for variables predicting chalkbrood infection (n=649).** | | | | | | | | | | | | | | | | | | | |
| --- | --- | --- | --- | --- | --- | --- | --- | --- | --- | --- | --- | --- | --- | --- | --- | --- | --- | --- | --- |
|  | **Observed** | |  | **Predicted** | | | | | |  | | | | |  | | |  |  |
|  |  | |  | No Chalkbrood | | | | Chalkbrood | | | | Percentage correct | | | | |  |  |  |
|  | No Chalkbrood | |  | 497 | | | | 0 | | | | 100 | | | | |  |  |  |
|  | Chalkbrood | |  | 152 | | | | 0 | | | | 0.0 | | | | |  |  |  |
| Overall Percentage | | |  |  | | |  | | | | | 76.6 | | | | |  |  |  |
| Nagelkerke R^2^ | |  | | |  |  | | | | |  | | 0.000 | | |  | | |  |
| **Variable** | | ***B*** | | | **SE *B*** | **Wald** | | | ***df*** | | | **P** | | **Odds Ratio** | | | | |  |
| HygStrict | | -0.045 | | | 0.322 | 0.019 | | | 1 | | | 0.890 | | 0.956 | | | | |  |
| HygLiberal | | -0.028 | | | 0.251 | 0.013 | | | 1 | | | 0.910 | | 0.972 | | | | |  |
| Constant | | -1.168 | | | 0.113 | 106.150 | | | 1 | | | 0.000 | | 0.311 | | | | |  |
| Dependent variable: chalkbrood presence. Predictor variables: Strict and liberal tests of hygienic behaviour | | | | | | | | | | | | | | | | | | |  |
